# Association between Taste Sensitivity and Self-Reported and Objective Measures of Salt Intake among Hypertensive and Normotensive Individuals

**DOI:** 10.5402/2013/301213

**Published:** 2012-10-24

**Authors:** Paula de Moura Piovesana, Karina de Lemos Sampaio, Maria Cecília B. J. Gallani

**Affiliations:** ^1^Faculty of Nursing, University of Campinas (UNICAMP), 13083-887 Campinas, SP, Brazil; ^2^Faculty of Food Engineering, UNICAMP, 13083-862 Campinas, SP, Brazil; ^3^Faculty of Nursing, Laval University, Quebec, QC, Canada G1V 0A6

## Abstract

This study investigated the gustatory threshold for salt and its relationship with dietary salt intake among hypertensive (*n* = 54) and normotensive (*n* = 54) subjects. Salt intake was evaluated through 24-hour urinary sodium excretion and self-reported measures (discretionary salt, Sodium- Food Frequence Questionnaire (Na-FFQ), and 24-hour recall). Detection and recognition thresholds were higher among hypertensive subjects, as well as the total sodium intake. Detection and recognition thresholds were positively related to discretionary salt and total intake of the group as whole. Hypertensive and normotensive subjects presented positive correlations between taste sensitivity and the different measures of salt intake. To conclude, a positive correlation exists between taste threshold and salt intake and both seem to be higher among hypertensive subjects. The combined use of methods of self-report and assessment of taste thresholds can be useful in health promotion and rehabilitation programs, by screening subjects at higher risk of elevated salt intake and the critical dietary behaviors to be targeted as well to evaluate the result of targeted interventions.

## 1. Introduction

Hypertension is considered an important public health problem due to its high prevalence worldwide and its continuous and independent linear correlation with cardiovascular risk. Indeed, this disease is considered one of the major risk factors for the majority of cardiovascular-related deaths [1].

The development and progression of hypertension depends on several factors, some of them intrinsic to the individual, such as the genetic profile. The complex interaction of these intrinsic factors with the environment, including here the health-related behaviors, is responsible for the magnitude of the clinical expression of the disease. Among the health-related behaviors, high sodium intake can be highlighted as an independent risk factor for increased risk of cardiovascular disease, especially hypertension [2–4]. Therefore, the reduction of dietary sodium intake is largely recognized and recommended as a necessary measure to prevent hypertension and to obtain a better control of the blood pressure levels [2, 5]. 

Besides these recommendations, salt intake remains elevated worldwide [5, 6]. As a complex behavior, the overall salt intake results from different sources. In developed countries, the main component of dietary salt is derived from industrialized foods, while for developing countries, the salt added during and after meal preparation has the most important contribution for the overall intake, as demonstrated in the studies conducted at general population [4, 7] and with hypertensive subjects [8, 9]. 

Salt intake is a nutritional behavior influenced by a multitude of factors. Cultural and socioeconomic factors in conjunction to intrinsic characteristics play an important role in the hedonistic enjoyment of eating [7]. This influence would be intermediated by the salty taste sensitivity, resulting in the modulation of the amount of salt intake. Reduced sensitivity would drive individuals to consume more salt until reach the salt concentration identified as pleasant. Furthermore, it is supposed that high salt intake gradually decreases salt taste perception and, consequently, increases the sodium concentration needed to trigger the detection and recognition of the salty taste, that is, detection and recognition thresholds [10]. 

Higher salt detection and recognition thresholds have been observed in subjects with hypertension or diabetes, elderly ones and those with high body mass index [11, 12]. Studies related to gustatory salt threshold in most cases have investigated its relationship with clinical profile and, less frequently, your association with sodium intake. One study [13] examined the relation between taste thresholds and dietary sodium intake using the urinary sodium excretion to estimate sodium consumption. It was observed that natriuresis was related to taste threshold. However, the sources of salt consumption vary widely and the measure of urinary sodium provides little information about the major sources of nutrient consumption [14]. On the other hand, the self-report methods have been increasingly recognized as a useful tool not only to quantify the salt intake, but also to identify different sources of salt contributing to the overall intake [15]. This precision is important because it provides valuable data on identification and understanding of the overall pattern of salt consumption in different populations. This information is helpful to health professionals in their clinical practice by guiding their educational interventions towards how best to reduce salt intake in specific groups or populations. 

Nonetheless, to our knowledge, up to now no study has evaluated taste threshold and its relationship with dietary salt measured by self-report methods. Considering previous reports in the literature pointing to lower levels of taste sensibility among hypertensive subjects and the paucity of data regarding the relation between intake and taste sensibility, this study was aimed at investigating, among hypertensive and normotensive individuals, the gustatory threshold for salt and its relationship with dietary sodium intake measured by an objective measure and self-report methods. 

## 2. Methods 

### 2.1. Participants

A total of 108 participants (54 hypertensive and 54 normotensive subjects) were recruited at outpatient clinics specialized in hypertension, one from a teaching hospital (29 hypertensive and 47 normotensive subjects) and the other from a basic health unit (25 hypertensive and 7 normotensive subjects), in Southeast Brazil. 

Inclusion criteria for the hypertensive group were age between 30–65 years, medical followup at least six months after the diagnosis of hypertension and be on a *stable regimen of antihypertensive therapy* for at least one month. 

Normotensive subjects were students, staff members, or employees, with 30–65 years of age and presented systolic and diastolic blood pressures below 130 and 85 mmHg, respectively, at a casual blood pressure measurement [17].

All participants had no history or evidence on physical examination of (1) taste and smell impairment, (2) diabetes mellitus, (3) neurological diseases (i.e., Parkinson's disease, stroke), (4) current or previous radiotherapy and chemotherapy treatments, (5) smoking (or nonsmoking for a period of less than one year), and (6) acute or chronic upper airway infection at the time of evaluation.

The study was approved by the local ethics committee and all eligible subjects were consecutively enrolled after giving written informed consent. 

### 2.2. Procedure

After their enrollment, participants were invited to schedule an interview on the same day or on subsequent days. During the interview, sociodemographic variables and anthropometric measures were first obtained and then, questions were targeted to salt intake, applying specific tools (discretionary salt, Na-FFQ, and 24-hour recall). Afterwards, patients were instructed to collect a 24-hour urine sample and to deliver it between the second and tenth days after the interview. Clinical data were obtained from the patient's chart. Participants were advised to collect a urine sample between Tuesdays and Fridays to prevent bias related to changes in weekend eating patterns.

### 2.3. Measures

#### 2.3.1. Measurements of Detection and Recognition Thresholds

For the evaluation of salt taste sensibility, after a review of the literature [18] it was adopted the procedure proposed by Nilsson [19] adapted by Hatae et al. [20] and described elsewhere [13, 21–24]. Participants were asked to attend without fasting but with abstention from eating or brushing their teeth at least one hour before the procedure, only being allowed to maintain water intake. The test was conducted in a private and quiet chamber without auditory stimulation. Eight solutions of sodium chloride (NaCl) diluted in distilled water at the following concentrations: 0.002, 0.004, 0.008, 0.016, 0.032, 0.064, 0.128, and 0.256 mol/L were used. The test was carried out by paired difference test. Individuals received a cup with 15 mL sample solution and another with distilled water, randomly. Cups were labeled with a 3-digit random number. Afterwards, they were asked to retain the sample solution in their mouth for a few seconds and then, to judge which of them had a taste sensation. Between the tests, the mouth was washed with distilled water, with 30 s intervals among successive tests. When the subject reported the taste sensation twice in the same concentration, he/she was given a solution of lower concentration until they failed to identify the taste. When the subject was not able to discriminate solutions, a next trial was conducted using an increased concentration of NaCl. The lowest concentration in which the individual felt stimulation three times was considered the detection salt taste threshold (DSTT). Afterwards, the concentration of NaCl was increased, following the same procedures, until the identification of the stimulus as a salty taste. This concentration was defined as the recognition salt taste threshold (RSTT) [20]. 

#### 2.3.2. Self-Reported Measures of Salt Intake

*Discretionary Salt*. Subjects were asked to rate their usual monthly amount of salt consumption and also to count the number of persons per household who had at least five meals per week at home, in order to correct the salt consumption per person. The USDA reference (1 gram of salt = 400 mg of sodium) was used to obtain the monthly and, afterwards, the daily sodium intake per person [6]. 

*24-h Recall*. This measure was assessed at an interview in which the participants were asked to describe, in as much detail as possible, all foods and beverages consumed over the past 24-hour period, including the usual portion size of these foods; they were asked to report these amounts using regionally typical utensil sizes as cups, glasses, spoons, slices, and servings [9]. 

*Sodium-Food Frequency Questionnaire (Na-FFQ)*. The Na-FFQ, originally called *Questionário de Frequência Alimentar de alimentos com alto teor de sódio* (QFASó) [9], developed and validated for evaluating sodium intake in low-income hypertensive Brazilian patients was used. It is a fifteen items questionnaire, consisting mostly of foods made in industry such as canned dairy and meat products, condiments, snacks, and fast food, regularly present in the nutritional practice of this population. Participants are asked how frequently each food was consumed during the last year, with responses ranging from 1 = never up to 7 = twice or more per day. For each food on the list, a Na-conversion factor was derived from food composition tables. The conversion factor was a number between 0.01 and 1.00, representing the amount of Na in 1 g of food. For each participant, the Na-content of the average portion consumed was calculated by multiplying the weight of the portion (in grams) by the conversion factor. Finally, the Na-milligrams in the usual serving size were multiplied by the frequency of intake and corrected for the frequency of monthly consumption (0 to score 1, representing never; until 60 to score 7, representing a daily frequency of twice or more). 

*Nutrient Database*. Sodium intakes from both dietary assessments were calculated by using data from the Nutwin database software developed by the Federal University of São Paulo [25].

#### 2.3.3. 24-Hour Urinary Sodium Excretion

Subjects were carefully instructed to collect all urinary volume during the 24-hour period and to drink water normally. Sodium excretion was measured by spectrophotometry and converted to mEq/L [4]. Sodium intake was estimated assuming that 1 mEq sodium reflects approximately 0.058 grams of salt intake [6].

## 3. Statistical Analysis 

Statistical analysis was performed with the SAS statistical software package release 9.1.3 (SAS Institute Inc., Cary, NC, USA) [26]. As the variables presented a nonnormal distribution, nonparametric tests were used for the inferential analysis. The chi-square and Mann-Whitney tests were used to test the association and to compare sociodemographic and clinical variables between groups. Mann-Whitney and the Spearman correlation tests were applied to compare salt intake and taste thresholds between groups and to evaluate the correlation between the different measures of salt consumption and taste thresholds. A power calculation revealed that a sample size of 90 subjects provided a power of 0.8 to detect a mean difference in recognition thresholds for sodium chloride of 0.046 mol/L between hypertensive and normotensive subjects (based on previous study the Spritzer, 1985), with a moderate effect size (*d* = 0.6) [16]. The sample was enlarged to 108 subjects, in order to circumvent possible losses. A *P* value less than 0.05 was considered significant.

## 4. Results

 The sociodemographic and clinical characteristics of the 108 patients are summarized in Table 1. The predominant group was female (85.0%), white (59.3%), aged 46.3 (±10.8) years, married (51.8%), with 10.8 (±5.5) years of schooling, and professionally active (64.9%). The group of hypertensive subjects presented a greater prevalence of male sex, non-Caucasians, and of individuals professionally not active, as well as higher levels of blood pressure, mean body mass index, and waist circumference, compared to the normotensive group. The mean length of hypertension diagnosis was 9.6 (±8.5) years. Hypertensive subjects took on average 1.7 ± 1.5 antihypertensive medications. From the whole sample, 40 subjects were treated with diuretics (38 among the hypertensive group, and two of the normotensive subjects). Among those on diuretic treatment, 32 were in use of thiazide diuretics, six on loop diuretics and two on potassium-sparing diuretics. 

### 4.1. Quantifying Sodium Taste Threshold and Salt Intake 

For the group-as-a-whole, the threshold for detection was 0.011 (±0.016) mol/L and for recognition, 0.020 (±0.031) (Table 2). Both thresholds were significantly higher for the group of hypertensive subjects (*P* = 0.001 and *P* = 0.0002, resp.).

 The result of the sum of the three methods (Na-FFQ, 24-h recall and discretionary salt) pointed to a total intake of 10.8 (±5.0) g salt/day, quite similar to that pointed by the biological measure of urinary sodium (10.3 ± 4.3 g). Discretionary salt was the most important source of salt intake, representing for the total sample around 60% of the overall intake. Sodium *in nature* (24-h recall) and the consumption of foods with high sodium content (Na-FFQ) contributed to 26% and 17%, respectively, to the total salt intake. The total intake given by the self-reported methods was significantly higher for hypertensive subjects (*P* = 0.046) as was the use of discretionary salt (*P* < 0.001), though intakes measured by Na-FFQ and 24-h recall were higher among normotensive individuals (*P* = 0.010 and *P* = 0.002, resp.). The measure of urinary sodium excretion was slightly more elevated among hypertensive subjects, but this difference was not statistically significant.

### 4.2. Correlating Sodium Taste Threshold and Salt Intake

Thresholds were strongly intercorrelated either considering the group-as-a-whole or the groups of hypertensive and normotensive subjects separately; however, they presented different patterns of correlation with measures of salt intake (Table 3).

The detection threshold in the whole group was positively correlated with the measures of discretionary salt (*r* = 0.20; *P* = 0.046) and total intake (*r* = 0.23; *P* = 0.015) and inversely correlated with 24-h recall (*r* = −0.20; *P* = 0.040). These correlations were not observed in the group of normotensive subjects. Positive correlation was observed between the detection threshold and the measure of Na-FFQ in hypertensive individuals (*r* = 0.28; *P* = 0.042). 

Regarding recognition threshold, correlations were found with discretionary salt and total intake for the group-as-a-whole (*r* = 0.21; *P* = 0.030 and *r* = 0.23; *P* = 0.017). However, analyzing the groups, there were no significant correlations within the hypertensive and for the normotensive groups; correlation was observed only for the Na-FFQ in normotensive subjects (*r* = 0.28; *P* = 0.043).

## 5. Discussion

This study aimed to analyze the salt intake and its relation with salt taste detection and recognition thresholds among hypertensive and normotensive subjects. Studies in the area have been directed to investigate the relationship between sodium intake and taste threshold with clinical profile, but there are few studies on the relationship between thresholds and different measures of sodium intake.

First, our study replicated findings in the literature regarding higher gustatory thresholds in hypertensive subjects in comparison with normotensive subjects [11, 24]. The coexistence of reduced sensitivity and genetically determined conditions, as hypertension and diabetes, has fostered the hypothesis of a genetic predetermination of salt perception. This rationale is reinforced by data pointing to higher detection and recognition thresholds in offspring of hypertensive patients and children whose parents had diabetes in comparison with normotensive children whose parents did not have hypertension [27]. 

We also observed that salt intake measured by self-reported methods was significantly higher among hypertensive subjects, reinforcing the rationale that an impaired salt perception is linked to an involuntary excess salt consumption. 

In fact, correlations were observed between threshold and consumption for the group-as-a-whole, considering the self-report measures. Both thresholds were positive and moderately correlated with total salt intake and the measure of discretionary salt, the main source contributing to the total sodium intake for this population. Moreover, consumption of foods with high salt content, given by the Na-FFQ, was related to the detection threshold in hypertensive subjects and to the recognition threshold in normotensive subjects. These data added the information that increments in salt intake are proportionally linked to a reduction in salt taste. 

The impact of this interaction is not negligible since it has been suggested that higher intakes of salt would trigger complex interrelated neurohormonal and volume changes as well as production of reactive oxygen species and oxidative stress contributing not only to the increase of blood pressure, but also to a defective insulin sensitivity and impaired glucose homeostasis [11, 28]. 

The urinary sodium excretion is considered the gold standard for analysis of salt intake. However it is known that this method presents a number of limitations as the variability of nutrients intake from day to day in individuals with a large intraindividual variation in salt consumption [14, 29]. Another limitation of the urinary sodium method is that there is no absolute guarantee that all 24-hour urinary volume has been collected. 

In contrast, the self-reported methods used in this study, even though limited by recall bias, subjectivity, and lack of high precision in quantification, represent mostly a pattern of overall consumption along a certain period of time and, in this sense, they could be more representative of a pattern of consumption than an isolated measure of urinary sodium. These observations can explain the correlations observed between thresholds and self-report salt consumption but not with natriuresis. 

The use of three self-report methods for analysis of sodium intake is justified to allow nutrient quantification from various sources: foods with high sodium content (including salty spices), sodium in nature, and salt added during and after preparation of the meal. Therefore, when considering the methods together, it is possible to estimate the overall consumption, and when methods are analyzed separately, it can be estimated the contribution of each source to the total consumption. As a consequence, it is possible to identify the greatest source contributing to the overall salt intake, which should be targeted for change in educational interventions aimed at reducing salt intake. In this study, as in previous studies in similar populations [4, 8, 9], the salt added to food during or after its preparation, that is, the discretionary salt, was appointed as a source of greater impact on total consumption.

In this sample, consumption of both groups exceeded the limits recommended for hypertensive and normotensive subjects, according to estimates of urinary sodium excretion and self-report methods. Whereas self-report consumption methods among normotensive subjects exceeded 1.6 times the recommended values for the general population (6 g/day), for the hypertensive patients, the rate was four times higher than the recommended limits (4 g/day). In fact, excessive salt consumption is a worldwide problem. Several initiatives are proposed against this problem, including governmental ones, emphasizing the benefits of reducing sodium intake [6, 30]. 

The public awareness about the benefits of reducing salt intake is important, but not enough. It is necessary to understand the factors that contribute to the excessive consumption, proposing strategies for effectively helping subjects to change their behavior. Making people aware of their own taste of salt along with the quantification and identification of the main sources of salt intake may be useful for educational purposes aimed at reducing salt intake. Additionally, the use of prospective measures of salt taste may be helpful for the individual to perceive changes in the ability to detect salt in foods and use it as a reinforcing strategy to further reductions in salt intake. 

This study present as limitations the subjectivity of the self-reported measures of salt intake and the concurrence of other factors that may interfere with taste perception tests, although the well-established exclusion criteria had been helpful to eliminate several bias. Furthermore, there is no clear cutoff value for the taste thresholds for salt, limiting some possibilities of analysis. Another limitation of this study was the impossibility to exclude patients using diuretics. There is a study demonstrating that for patients taking loop diuretics the urinary sodium is not a reliable measure to estimate salt intake [31]. But we do not have clues about the effect of such drugs on the taste threshold for salt as well as the effect of other diuretics on the urinary sodium excretion. Finally, considering the relative small sample size in the split groups of hypertensive and normotensive subjects, as well as the impossibility to age match the groups it is recommended that findings are corroborated in a study involving a larger study population. 

## 6. Conclusions

 Our data indicated that salt intake and taste thresholds (detecting and recognizing) for sodium were higher for the hypertensive subjects. Taste thresholds were positively related to measures of self-reported sodium intake.

Considering the panorama of excess sodium intake and consumption reduction campaigns, the use of self-report and threshold assessment methods can be used not only in designing targeted health education interventions for the hypertensive subjects but also for the general population as preventive therapy in controlling cardiovascular diseases.

## Figures and Tables

**Table 1:** Sociodemographic and clinical profile.

	Total Mean (SD)*	Normotensive Mean (SD)	Hypertensive Mean (SD)	*P* value^†^
Age (years)	46.3 (10.8)	39.0 (8.4)	53.6 (7.5)	<0.001
Schooling (years)	10.8 (5.5)	14.6 (3.1)	7.1 (4.8)	<0.001
Diagnosis length	—	—	9.6 (8.5)	—
SBP^¶^ mmHg	126.9 (23.6)	112.2 (9.4)	141.9 (24.2)	<0.001
DBP^§^ mmHg	78.5 (13.8)	70.9 (8.8)	86.2 (13.7)	<0.001
MBP^‡^ mmHg	94.6 (16.0)	84.9 (8.5)	104.9 (15.8)	<0.001
PP** mmHg	48.4 (16.1)	41.2 (7.0)	56.2 (19.0)	<0.001
BMI^††^ Kg/m^2^	28.1 (5.6)	25.2 (4.4)	31.0 (5.2)	<0.001
WC^¶¶^ cm	93.3 (11.4)	87.7 (10.4)	99.0 (9.5)	<0.001

	*n* (%)	*n* (%)	*n* (%)	*P* value^§§^

Gender				
Female	92 (85.2%)	50 (92.6%)	42 (77.8%)	0.404
Skin color				
White	64 (59.3%)	42 (77.8%)	23 (42.6%)	0.018
Marital Status				
With partner	67 (62.1%)	29 (53.8%)	38 (70.4%)	0.272
Employment				
Active	73 (67.6%)	50 (92.6%)	23 (42.6%)	0.002
Nonactive	19 (17.6%)	3 (5.6%)	16 (29.6%)	0.005
Housewife	16 (14.8%)	1 (1.8%)	15 (27.8%)	<0.001
Monthly family income***				
U$ 0–1620.8	55 (55.6%)	15 (31.9%)	40 (76.9%)	0.001
>U$ 1620.8	44 (44.4%)	32 (68.1%)	12 (23.1%)	0.002

^*∗*^SD: standard deviation; ^†^Mann-Whitney test; ^¶^SBP: systolic blood pressure, ^§^DBP: diastolic blood pressure; ^‡^MBP: mean blood pressure; **PP: pulse pressure = [SBP − DBP]; ^††^BMI: body mass index = [weigh/height^2^]; ^¶¶^WC: waist circumference [16]; ^§§^Chi-square; ^‡‡^Missing: 2 normotensive; ***Missing: 7 normotensive and 3 hypertensive subjects.

**Table 2:** Salt intake and taste threshold.

	Total		Normotensive subjects		Hypertensive subjects		*P* value^¶^
	Mean (SD)*	Median (IQR)^†^	Mean (SD)	Median (IQR)	Mean (SD)	Median (IQR)
Detection threshold (mol/L)	0.011 (0.016)	0.008 (0.008)	0.008 (0.009)	0.004 (0.004)	0.015 (0.002)	0.008 (0.009)	0.001
Recognition threshold (mol/L)	0.020 (0.031)	0.008 (0.012)	0.013 (0.017)	0.008 (0.008)	0.027 (0.016)	0.016 (0.240)	<0.001
Self-report methods							
FFQ (g salt/day)^‡^	1.8 (1.8)	1.3 (1.6)	2.2 (1.9)	1.6 (2.1)	1.5 (1.6)	0.9 (1.4)	0.010
Discretionary salt (g salt/day)	6.3 (5.4)	5.4 (5.4)	4.3 (2.8)	4.0 (4.8)	8.2 (6.4)	6.3 (5.9)	<0.001
24 h recall (g salt/day)	2.9 (1.6)	2.7 (2.2)	3.4 (1.7)	3.1 (3.6)	2.4 (1.4)	2.1 (1.7)	0.002
Total intake (g salt/day)**	10.8 (5.0)	9.7 (4.7)	9.6 (3.1)	9.5 (3.9)	12.1 (6.2)	10.5 (4.9)	0.046
24-hour urinary sodium excretion^§^							
mEq/24 h	177.1 (65.8)	170.5 (71.5)	188.5 (73.6)	175.1 (73.4)	165.7 (55.4)	169.0 (63.0)	0.175
Converted to g salt/day	10.3 (3.8)	9.9 (4.2)	9.8 (4.5)	9.8 (3.6)	10.9 (4.3)	10.1 (4.3)	0.175

^∗^SD: standard deviation; ^†^IQR: interquartile range; ^¶^Mann-Whitney test; ^§^Missing: 3 normotensive and 3 hypertensive subjects; ^‡^FFQ: food frequency questionnaire. **Total intake: sum of FFQ, discretionary salt, and 24-h recall.

**Table 3:** Analysis of correlation* between sodium consumption and taste thresholds.

	FFQ^†^	*Per capita * ^¶^	24-h recall	Total intake^§^	Urinary sodium^‡^
Detection threshold					
Total sample	0.16 (0.099)	0.20 (0.046)	−0.20 (0.040)	0.23 (0.015)	−0.04 (0.654)
Hypertensive subjects	0.28 (0.042)	0.12 (0.382)	−0.09 (0.523)	0.20 (0.151)	−0.11 (0.424)
Normotensive subjects	0.21 (0.124)	0.07 (0.645)	−0.14 (0.315)	0.16 (0.252)	−0.09 (0.511)
Recognition threshold					
Total sample	0.1 (0.287)	0.21 (0.030)	−0.15 (0.112)	0.23 (0.017)	−0.06 (0.578)
Hypertensive subjects	0.16 (0.244)	0.17 (0.217)	−0.04 (0.752)	0.2 (0.155)	−0.06 (0.655)
Normotensive subjects	0.28 (0.043)	−0.03 (0.842)	−0.06 (0.666)	0.1 (0.467)	−0.19 (0.176)

^∗^Spearman (*P* value); ^†^FFQ: food frequency questionnaire; ^¶^ *per capita*: discretionary salt; ^§^total intake: sum of FFQ, *per capita* and 24-h recall, ^‡^Missing: 3 normotensive and 3 hypertensive subjects.
